# Preoperative assessment of cervical lymph node metastases in patients with papillary thyroid carcinoma: Incremental diagnostic value of dual-energy CT combined with ultrasound

**DOI:** 10.1371/journal.pone.0261233

**Published:** 2021-12-13

**Authors:** Jimin Yoon, Yangsean Choi, Jinhee Jang, Na-Young Shin, Kook-Jin Ahn, Bum-soo Kim

**Affiliations:** Department of Radiology, Seoul St. Mary’s Hospital, College of Medicine, The Catholic University of Korea, Seoul, Republic of Korea; Jr., Brigham and Women’s Hospital, Harvard Medical School, UNITED STATES

## Abstract

**Purpose:**

To determine whether dual-energy CT (DECT) has incremental diagnostic value when combined with ultrasound (US) in the diagnosis of metastatic cervical lymph nodes (LNs) in patients with papillary thyroid carcinoma (PTC).

**Methods:**

This was a single-center retrospective cohort study of patients diagnosed with PTC between October 2019 and August 2020. US features of LNs to include hyperechogenicity, round shape, microcalcification, cystic component, and homogeneous/peripheral vascularity were considered suggestive of metastasis. The HU of arterial phase (HU_arterial_) and DECT-derived CT images [contrast media (CM) and areas under the 100 keV monoenergetic curve (AUC_100keV_)] were measured. Effective atomic numbers (Z_eff_), iodine concentration (mg/mL), and slope of the HU curve (λ_HU_) were also obtained. The values for metastatic and benign LNs were compared using Student’s *t*-test with false-discovery correction. Logistic regression with areas under the receiver operating characteristic curves (AUCs) were performed for predicting metastatic LNs.

**Results:**

A total of 102 patients were included (49 metastatic and 53 benign LNs; mean age, 46±15 years). Metastatic LNs showed significantly higher values for HU_arterial_, CM, Z_eff_, λ_HU_, AUC_100keV_, and iodine concentration (all, *P* = 0.001). In logistic regression, the HU_arterial_ demonstrated the highest AUC (0.824; 95% confidence interval [CI], 0.751–0.897), followed by CM HU (0.762; 95% CI, 0.679–0.846). Combination of DECT parameters with US features improved the AUC from 0.890 to 0.941.

**Conclusion:**

Compared to US features alone, combination with DECT-derived quantitative parameters improved diagnostic performance in predicting metastatic cervical LNs in patients with PTC.

## Introduction

Thyroid cancer is the most common head and neck malignancy, and its incidence is increasing with the widespread application of ultrasound (US) screening and needle biopsy of suspicious nodules [1]. Among the various subtypes, papillary thyroid carcinoma (PTC) accounts for more than 80% of all thyroid malignancies. Of importance, patients with PTC have high rates of lymph node (LN) metastasis at an early stage [2]. In fact, the incidence of LN metastasis in PTC ranges from 30% to 90% based on the literature [3, 4]. In patients with PTC, metastases to cervical LNs at presentation are associated with local recurrence and cancer-related mortality [5, 6]. Therefore, accurate preoperative diagnosis of cervical LN metastasis is clinically relevant for optimizing treatment planning and improving patient prognosis.

Currently, US is the modality of choice for preoperative screening in patients with PTC; however, several studies have verified the usefulness of neck CT as an alternative [7] or complementary [8] imaging modality in detecting cervical LN metastasis. Furthermore, with the recent advent of dual-energy CT (DECT), anticipation of a diagnostic role of CT is increasing in various fields [9]. In brief, DECT utilizes two X-ray tubes with different energy levels, which helps to overcome the inherent limitation of conventional single-source CT in material-specific differentiation of soft tissues. Moreover, quantitative data for specific materials such as iodine and calcium can be acquired via DECT. These additional capabilities of DECT may allow more accurate differentiation between benign and malignant cervical LNs.

Several previous studies demonstrated the role of DECT in detecting and characterizing the cervical LN metastasis of PTC. In a previous study by Liu *et al*. [10], the single best quantitative DECT parameter was found to be the slope of the spectral HU curve (λ_HU_) in the venous phase. The current study applied additional DECT parameters, including effective atomic number (Z_eff_) and iodine concentration (IC, mg/mL), in predicting metastatic cervical LNs in patients with PTC. In doing so, we attempted to assess whether DECT-derived parameters add diagnostic benefits for cervical LNs when combined with the presence of suspicious US features (i.e. hyperechogenicity, microcalcification, round shape, cystic component, and homogeneous/peripheral vascularity) [11].

Therefore, the purpose of this study was to determine whether DECT parameters add diagnostic value to US in differentiating malignant from benign cervical LNs in patients with PTC.

## Materials and methods

This study was approved by the institutional review board of Seoul St. Mary’s Hospital with a waiver of informed consent due to retrospective nature of study (KC20RISI0466). The current study adhered to the Strengthening the Reporting of Observational Studies in Epidemiology (STROBE) statement [12].

### Study population

This single-center retrospective cohort study was approved by the institutional review board of our institution, and the requirement for informed consent was waived. Between October 2019 and August 2020, records of 108 consecutive patients with PTC who had undergone thyroid US and DECT were reviewed. The inclusion criteria for patients were: 1) PTC diagnosis; 2) available DECT of the neck; 3) pathological diagnosis for LN of interest; 4) interval between US and DECT of less than six months; and 5) one LN of interest per one cervical level. US-guided fine-needle aspiration or core-needle biopsies were performed on patients with suspected cervical LN metastases. Prior to surgery, all patients underwent CT imaging of the neck.

### Ultrasonography

All US images were obtained by either radiologists in training under faculty supervision or by board-certified radiologists. A linear transducer (7.5−15 MHz) of one of two US scanners (iU22, Philips Healthcare, Andover, MA, USA; Aplio i700, Canon Medical Systems Corporation, Tustin, CA, USA) were used. US images of both thyroid lobes, bilateral cervical levels from I to VI, and the supraclavicular areas were acquired. The US findings suggestive of metastatic cervical LN were as follows: microcalcification, cystic changes, abnormal vascularity (homogeneous or peripheral pattern), and focal or diffuse hyperechogenicity [11]. Any cervical LN showing a suspicious feature underwent fine-needle aspiration or core-needle biopsy by board-certified radiologists prior to surgery.

### CT acquisition protocol

All CT images were acquired using two third-generation dual-source CT scanners (Somatom Force, Siemens Healthineers, Issaquah, WA, USA). CT scanning was initiated at the aorticopulmonary window and ended at the skull base. The acquisition protocol included contrast-enhanced axial images after intravenous administration of 90 mL of iodinated contrast medium (Optiray, 100 mL, Reyon Pharmaceutical, South Korea) per antecubital vein at a rate of 4 mL/s using an automated injector, followed by flushing with 30 mL of normal saline. Our institutional protocol included arterial phase CT scans (at 25-s delay) based on prior publications demonstrating improved diagnostic performance over venous phase CT scans [13, 14]. The DECT scans were acquired in the venous phase (at 80-s delay). All CT images were reconstructed into coronal images. The scanning parameters were as follows: detector collimations, 128 × 0.6 mm; pitch, 0.6; gantry rotation time, 1 s/rotation; matrix, 256 × 256; tube current, automated modulation (Care Dose 4D; Siemens Healthineers); tube voltage, 80/140 Sn kVp; filter back projection with soft tissue kernel (B40f); and slice thickness, 3 mm. Radiation exposure was evaluated using CT dose index volume (CTDIvol) and dose-length product (DLP). The median time interval between US and CT examinations was 15.7 days (interquartile range, 1 to 16 days).

### Image analysis

Cervical levels were determined based on the American Joint Committee on Cancer level system [15]. On axial CT images, one radiologist with 6 years of experience in thyroid radiology drew circular ROIs on LNs that previously underwent US-guided biopsies. ROIs over the left internal carotid artery were also drawn on the same CT images. The radiologist was blinded to the pathologic reports of individual patients. All ROIs were reviewed by a second radiologist with 9 years of experience in thyroid radiology. Any discrepancies were resolved by consensus. To evaluate for selection bias in ROI measurements, three circular ROIs were drawn (top, middle, and bottom) in a subset of data (65 available dual-energy CT images) (S1 Fig). All image analyses were performed using *syngo*.via software VA30 (Siemens Healthineers).

HU_arterial_ and DECT-derived parameters, which included contrast medium (CM)—the relative HU attributable to contrast medium—and areas under the 100 keV monoenergetic curve (AUC_100keV_), were normalized with reference to that of the left internal carotid artery. Effective atomic numbers (Z_eff_), IC, and the slope of the HU curve (λ_HU_)—calculated as the difference between HU at 40 keV and that at 70 keV divided by the energy difference (30 keV) [10]—were also obtained and normalized.

Additionally, the conventional neck CT images were independently visually assessed by two blinded radiologists. The degree of enhancement, pattern of enhancement, and presence of cystic changes and calcification within the lesion were recorded. The degree of enhancement was assessed in the arterial phase and was graded as *none*, *mild*, *moderate*, and *strong* enhancement. Also, the US images taken during fine-needle aspiration or core-needle biopsy were reviewed. Any malignant LN features—hyperechogenicity, round shape, microcalcification, cystic changes, and homogeneous/peripheral vascularity on Doppler US—were recorded.

### Statistical analysis

The patients were divided into *metastatic* and *benign* LN groups according to the pathological diagnosis from either fine-needle aspiration or core-needle biopsy. Continuous and categorical variables were compared via the Wilcoxon rank-sum test and Fisher’s exact test, respectively. The interrater agreement on conventional CT and US findings between the two radiologists was assessed via Cohen’s kappa. The data acquired from the single- and three-ROI methods were compared via independent *t*-test. Furthermore, intraclass correlation coefficients of DECT parameters acquired from the two different ROI measurement methods were calculated. To determine the associations of individual CT parameters with metastatic LNs, univariate logistic regression was initially performed. Variables with statistically significant differences were then subjected to multivariate logistic regression. ROC curves were plotted using the significant variables from US images, DECT parameters, and both combined. Statistical significance was set at *P*<0.05. All statistical analyses were performed using R statistical software (v. 3.6.1, R Foundation for Statistical Computing, Vienna, Austria).

## Results

### Patient characteristics

**Fig 1:**
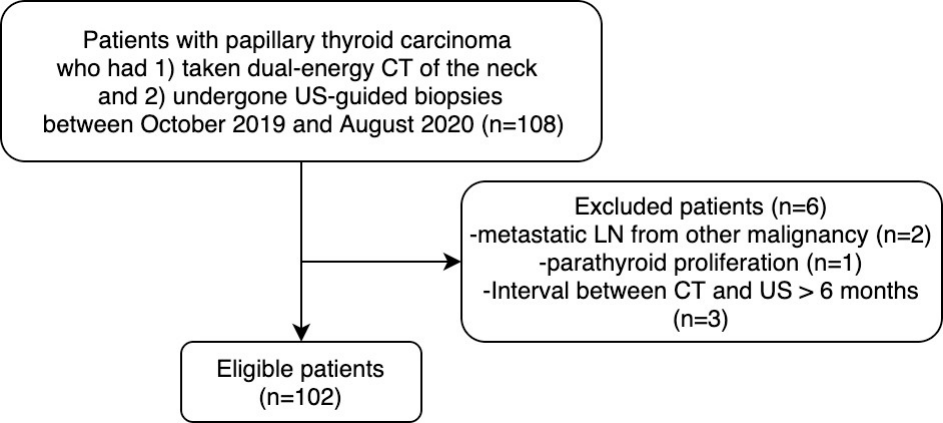
Flow chart illustrating patient selection process.

A total of 108 patients were retrospectively screened. Among them, 6 patients were excluded due to a) LN metastasis from other malignancy (n = 3) and b) pathological diagnosis as parathyroid lesion (n = 3). A flow diagram of the patient selection process is depicted in Fig 1.

The characteristics of included patients are summarized in Table 1. Of 102 eligible patients, 70 were female (68.6%), and mean age was 46 ± 15 years. All included patients had underlying PTC with US-guided fine-needle aspiration or core-needle biopsy of a cervical LN performed. Among the 102 patients, 53 (52%) and 49 (48%) were found to have benign and metastatic LNs, respectively. The locations of examined LNs varied from cervical level II to level VI and the supraclavicular area; the most frequent locations were cervical levels III (32.4%, 33/102) and IV (31.4%, 32/102). Among 16 patients with more than one biopsied LN, none had LNs in the same cervical levels, which allowed clear radiological-pathological correlation. A detailed distribution of all included LNs is listed in Table 1. The mean CTDI volume for dual-energy CT of the neck was 26.4 ± 3.6 mGy, and DLP was 717.7 ± 107.5 mGy × cm.

**Table 1:** Baseline characteristics of study population.

Variable	n = 102
Sex, female n(%)	70 (68.6)
Age, mean±SD	46±15
Size of LN, mean±SD (mm)	7.9±3.9
Pathologic diagnosis	
Benign	53 (52)
Metastatic	49 (48)
Cervical level of LN	
II	15 (14.7)
III	33 (32.4)
IV	32 (31.4)
V	4 (3.9)
VI	11 (10.8)
Supraclavicular	6 (5.9)
Strap muscle	1 (1)
CTDI volume (mGy), mean±SD	26.4±3.6
DLP (mGy×cm), mean±SD	717.7±107.5

CTDI = computer tomography dose index; DLP = dose-length product; LN = lymph node; mGy = milligray; SD, standard deviation

### Comparison of measurements between metastatic and benign lymph nodes

**Fig 2:**
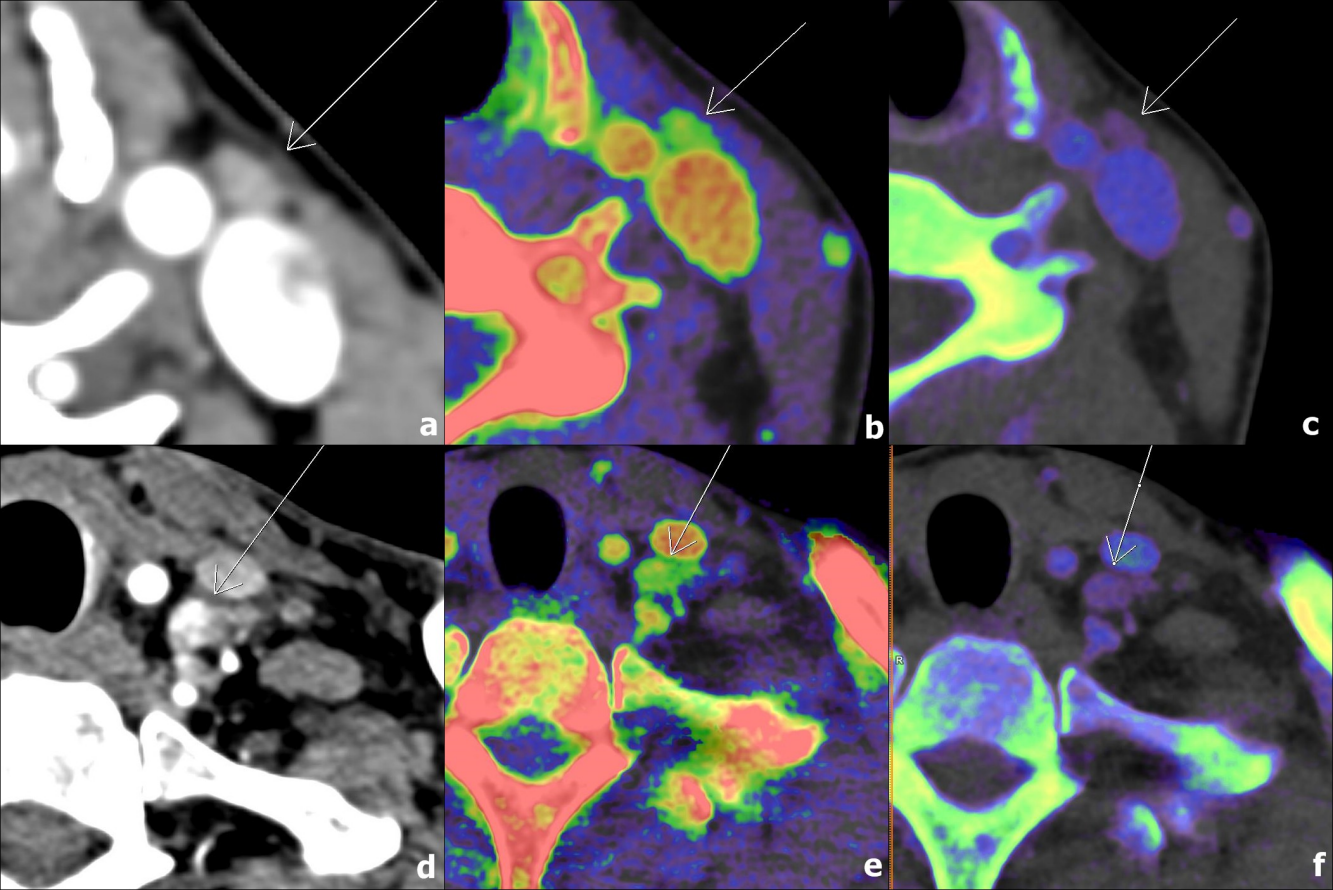
Axial CT images of arterial phase (a, d), DECT-derived contrast-enhancement (b, e), and Rho/Z (c, f) maps of two patients with papillary thyroid carcinoma. A 47-year-old female patient with a benign lymph node at left cervical level II (a, b, c; AUC_100keV_ = 36.7, HU_arterial_ = 0.32, Z_eff_ = 0.91, CM = 0.58, IC = 0.56, λ_HU_ = 4.7), and a 53-year-old male patient with a metastatic lymph node at left cervical level IV (d, e, f; AUC_100keV_ = 43.5, HU_arterial_ = 0.41, Z_eff_ = 0.93, CM = 0.62, IC = 0.58, λ_HU_ = 5.6).

Representative CT images of cervical LNs are shown in Fig 2. The results for parameters derived from conventional CT, US, and DECT for the benign and metastatic groups are shown in Table 2. There were no statistically significant differences based on sex and age between the two groups.

**Table 2:** Comparison of conventional and dual-energy CT findings of benign and metastatic lymph nodes.

Clinical variables	Benign (n = 68)	Metastatic (n = 65)	*P* ^a^
Age, mean±SD	46.7±13.4	42.8±16.2	0.136
Sex, female, n(%)	50 (73.5)	43 (66.2)	0.46
Conventional CT findings			
Degree of enhancement, n(%)			<0.001
None	0 (0)	1 (1.5)	
Mild	36 (52.9)	11 (16.9)	
Moderate	28 (41.2)	14 (21.5)	
Strong	4 (5.9)	39 (60)	
Pattern of enhancement, n(%)			0.018
Homogeneous	39 (57.4)	22 (33.8)	
Heterogeneous	29 (42.6)	43 (66.2)	
Cystic component, present n(%)	0 (0)	9 (13.8)	0.005
Calcification, present n(%)	2 (2.9)	11 (16.9)	0.015
Conventional US findings, present n(%)			
Hyperechogenicity	15 (22.1)	47 (72.3)	0.003
Round shape	17 (25)	35 (53.8)	0.005
Microcalcification	6 (8.8)	37 (56.9)	0.003
Cystic component	0 (0)	6 (9.2)	0.038
Homogeneous or peripheral vascularity	14 (20.6)	21 (32.3)	0.227
Dual-energy CT findings, mean±SD (HU)			
HU_arterial_	0.25±0.08	0.37±0.11	0.001
CM	0.37±0.13	0.53±0.21	0.001
IC (mg/ml)	0.36±0.14	0.51±0.2	0.001
λ_HU_	2.96±1.22	4.11±1.65	0.001
Z_eff_	0.88±0.04	0.91±0.04	0.001
AUC_100KeV_	31.5±7.7	39.1±9.6	0.001

^a^After false discovery rate correction

AUC_100KeV_ = area under the 100 KeV monoenergetic curve; CM = contrast media; HU_arterial_ = HU measured on arterial phase; IC = iodine concentration; SD = standard deviation; Z_eff_ = effective atomic number; λ_HU_ = slope of the HU curve

The interrater agreement for conventional CT and US findings was excellent (kappa range, 0.735–0.937) (Table 3). There were no significant differences in any DECT-derived parameters between the two ROI measurement methods (*P* = 0.267–0.999) (S1 Table); the intraclass correlation coefficients were excellent (S2 Table). The conventional CT findings showed significant differences between the benign and metastatic LN groups. More metastatic LNs showed strong arterial enhancement than did benign LNs (*P*<0.001). The metastatic LNs showed a higher frequency of heterogeneous enhancement (*P* = 0.018). Calcifications and cystic changes within the LN were more prevalent in the metastatic LN group (*P* = 0.005 and 0.015, respectively).

**Table 3:** Interrater agreement of conventional CT and US findings.

Conventional CT findings	κ*
Degree of enhancement	0.846
Pattern of enhancement	0.781
Presence of cystic component	0.937
Presence of calcification	0.857
Conventional US findings	
Hyperechogenicity	0.748
Round shape	0.767
Microcalcification	0.892
Cystic component	0.735
Homogeneous or peripheral vascularity	0.878

*Cohen’s kappa between two raters; all *P*-values were <0.001

The results for malignant US features in both groups are shown in Table 2. The frequencies of hyperechogenicity and round shape were higher in the metastatic LN group (*P* = 0.003 and 0.005, respectively). Consistent with conventional CT findings, the presence of cystic changes and microcalcifications was higher in the metastatic LN group (*P* = 0.038 and 0.003, respectively). Lastly, peripheral or homogeneous vascularity on Doppler US was more common in the metastatic LN group, but without statistical significance (*P* = 0.227). All measurements of DECT-derived parameters (i.e. HU_arterial_, CM, IC, λ_HU_, Z_eff_, and AUC_100keV_) were significantly higher in the metastatic LN group (all, *P* = 0.001; Table 2). Representative spectral HU curves for benign and metastatic LNs are shown in S2 Fig.

### Diagnostic performances in predicting metastatic lymph nodes

The AUC value, sensitivity, specificity, positive predictive value, and negative predictive value for each quantitative DECT parameter for differentiating metastatic from benign LNs are shown in Table 4. The HU_arterial_ showed the highest AUC value of 0.824 (95% CI, 0.751–0.897). Other quantitative parameters also showed good diagnostic performances with AUC values ranging between 0.699 and 0.754.

**Table 4:** Predictive performance of conventional and dual-energy CT parameters.

Parameters	AUC	Sensitivity (%)	Specificity (%)	PPV (%)	NPV (%)
HU_arterial_	0.824 (0.751, 0.897)	71 (58, 83)	93 (82, 99)	91 (80, 98)	77 (70, 85)
Z_eff_	0.726 (0.638, 0.814)	63 (40, 86)	81 (56, 96)	76 (64, 90)	70 (61, 82)
IC (mg/ml)	0.727 (0.638, 0.815)	65 (45, 85)	79 (56, 94)	75 (63, 90)	70 (63, 81)
λ_HU_	0.699 (0.609, 0.789)	69 (34, 83)	71 (56, 97)	70 (61, 91)	69 (60, 80)
CM	0.754 (0.668, 0.840)	66 (48, 82)	84 (71, 97)	80 (70, 95)	72 (65, 82)
AUC_100KeV_	0.747 (0.661, 0.834)	62 (43, 83)	85 (63, 97)	80 (67, 94)	70 (63, 80)

*Data in parentheses are 95% confidence intervals

AUC_100KeV_ = area under the 100 KeV monoenergetic curve; HU_arterial_ = HU measured on arterial phase; IC = iodine concentration; SD = standard deviation; Z_eff_ = effective atomic number; λ_HU_ = slope of the HU curve; PPV = positive predictive value; NPV = negative predictive value

**Fig 3:**
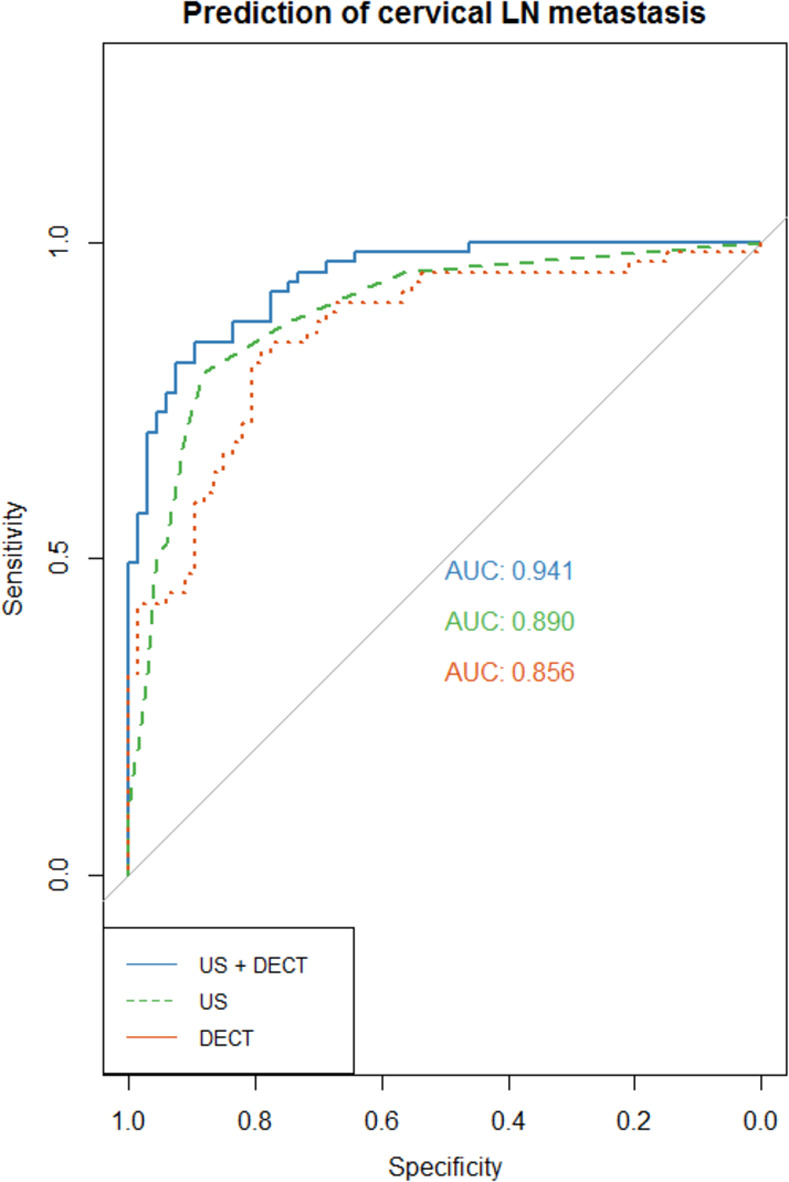
Receiver operating characteristic curves for diagnostic performance in predicting metastatic LNs based on DECT parameters alone (HU_arterial_, IC, CM, Z_eff_, AUC_100keV_) and in combination with suspicious features on US (hyperechogenicity, round shape, cystic component).

The diagnostic performances of US, DECT, and US combined with DECT in identifying metastatic LNs are displayed as ROC curves in Fig 3. The predictive performance of US with DECT (AUC = 0.941) was higher than that of either US (AUC = 0.890) or DECT (AUC = 0.856) alone.

## Discussion

In this study, we demonstrated the potential of DECT-derived quantitative parameters in differentiating metastatic from benign LNs in patients with PTC. In particular, HU_arterial_ showed the highest diagnostic performance. DECT-derived parameters including CM, AUC_100keV_, Z_eff_, and λ_HU_ also showed good performances (AUC range, 0.699–0.754). Compared to US alone (AUC = 0.890), the combination of US and DECT parameters increased the diagnostic performance in predicting metastatic cervical LNs (AUC = 0.941).

Similar work by Liu *et al*. demonstrated that the slope of the HU curve had significantly higher accuracy in identifying metastatic cervical LNs in PTC patients [10]. The main difference between our study and theirs is the DECT acquisition method—the current study used third-generation DECT scanners that applied dual-source X-ray tubes with tin filtration, whereas Liu *et al*. used DECT based on rapid switching of high and low tube voltages. Additionally, the current study selected cervical LNs that were targeted based on US-guided biopsy results, which allowed precise radiological-pathological correlation.

The potential benefit of DECT over conventional CT lies in the higher number of quantitative parameters for tissue characterization using DECT. In conventional contrast-enhanced CT, the mean HUs (i.e. CT numbers) are the results of combinations of the degree of iodine enhancement and the HUs of the underlying tissue. Thus, quantification of either HUs or IC in areas with mixed tissue types could be difficult. In DECT, IC can be quantified as iodine uptake per unit volume via iodine-based material decomposition imaging [14], which would be a more accurate representation of the true IC than HU. The third-generation DECT scanner used in this study was proven to accurately quantify IC [16]. Indeed, IC demonstrated good diagnostic performance in differentiating benign from malignant cervical LNs.

Currently, US is the modality of choice for preoperative imaging of thyroid diseases. US features of malignant thyroid nodules are defined by several guidelines [11, 17]. However, the role of neck CT in patients with thyroid cancer has drawn the interest of researchers recently [7, 8, 18]. A previous study showed acceptable performance of conventional CT in detecting metastatic LNs in thyroid cancer patients [7]. Another study demonstrated the complementary role of conventional CT in increasing the diagnostic confidence level for indeterminate LNs on US [18], which is consistent with the current study’s findings.

CT has an advantage over US in that CT provides objective images, whereas US is an operator-dependent modality. Furthermore, CT provides more comprehensive anatomical information, allowing visualization of deeply situated LNs—such as substernal or mediastinal LNs—that might otherwise be invisible on US due to its inherent limitation caused by disturbed ultrasonic propagation [19]. Therefore, employing both imaging modalities for the evaluation of metastatic cervical LNs would provide additional diagnostic confidence, as previously investigated in a relevant study [20].

It is noteworthy that HU_arterial_ showed the best diagnostic performance among various CT measurements. This is in accordance with previous works that demonstrated good performance of arterial phase CT in detecting metastatic cervical LNs in thyroid cancer patients [7, 21]. We assume that the arterial enhancement of metastatic LNs might be associated with tumor angiogenesis, which would induce recruitment of capsular vessels [21], whereas benign LNs would be supplied mainly from hilar vessels [22].

There are a few limitations in this study. First, due to the retrospective study design, the possibility of selection bias exists. However, the patients were included according to the predefined selection criteria in a consecutive time frame, which would have minimized selection bias. Second, exposure to ionizing radiation is inevitable with CT scans. However, CT has an advantage over US in that it allows comprehensive evaluation of the entire neck, including the lower cervical levels of VI and VII and the upper mediastinum. In this regard, CT scans must be considered complementary—and not an alternative to—US in assessing cervical LN metastasis [8, 18]. Finally, the results obtained from this single-center study might not be generalizable to other institutions with different imaging protocols.

In conclusion, DECT-derived quantitative parameters demonstrated good diagnostic performances in discriminating metastatic from benign cervical LNs in patients with PTC. When combined with US, DECT-derived parameters could serve a complementary role in discriminating metastatic cervical LNs in equivocal cases.

## Supporting information

S1 Fig Representative CT images of a 34-year-old female patient with a metastatic cervical lymph node in right cervical level IV. Three circular regions of interests were drawn from upper (a), middle (b), and lower (c) slices.
(TIFF)

S2 Fig Two spectral HU curves of a benign (left) and metastatic (right) LN. (TIF)

S1 Table Comparison of DECT-derived parameters between the two methods of ROI measurements. (DOCX)

S2 Table Intraclass correlation coefficients of DECT-derived parameters acquired by two methods of ROI measurements. (DOCX)

## Abbreviations

λ_HU_: slope of the HU curve
AUC_100keV_: area under the 100 keV monoenergetic curve
CM: contrast media
CTDI: computed tomography dose index
DECT: dual-energy CT
DLP: dose-length product
HU_arterial_: Hounsfield unit on arterial phase
IC: iodine concentration
LN: lymph node
PTC: papillary thyroid carcinoma
Z_eff_: effective atomic number
