# Supplementary figures and images for: Preoperative assessment of cervical lymph node metastases in patients with papillary thyroid carcinoma: Incremental diagnostic value of dual-energy CT combined with ultrasound
Source: PLoS One. 2021 Dec 13;16(12):e0261233. doi: 10.1371/journal.pone.0261233 (PMC8668122; doi:10.1371/journal.pone.0261233)

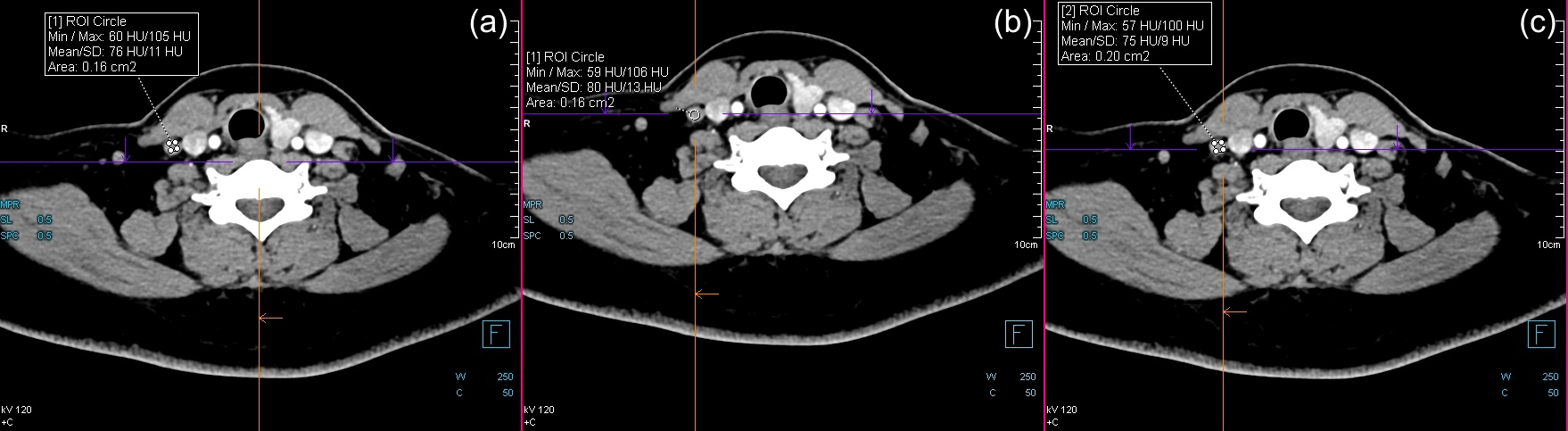

Supplement: S1 Fig — Three circular regions of interests were drawn from upper (a), middle (b), and lower (c) slices. (TIFF) [file pone.0261233.s001.tiff]

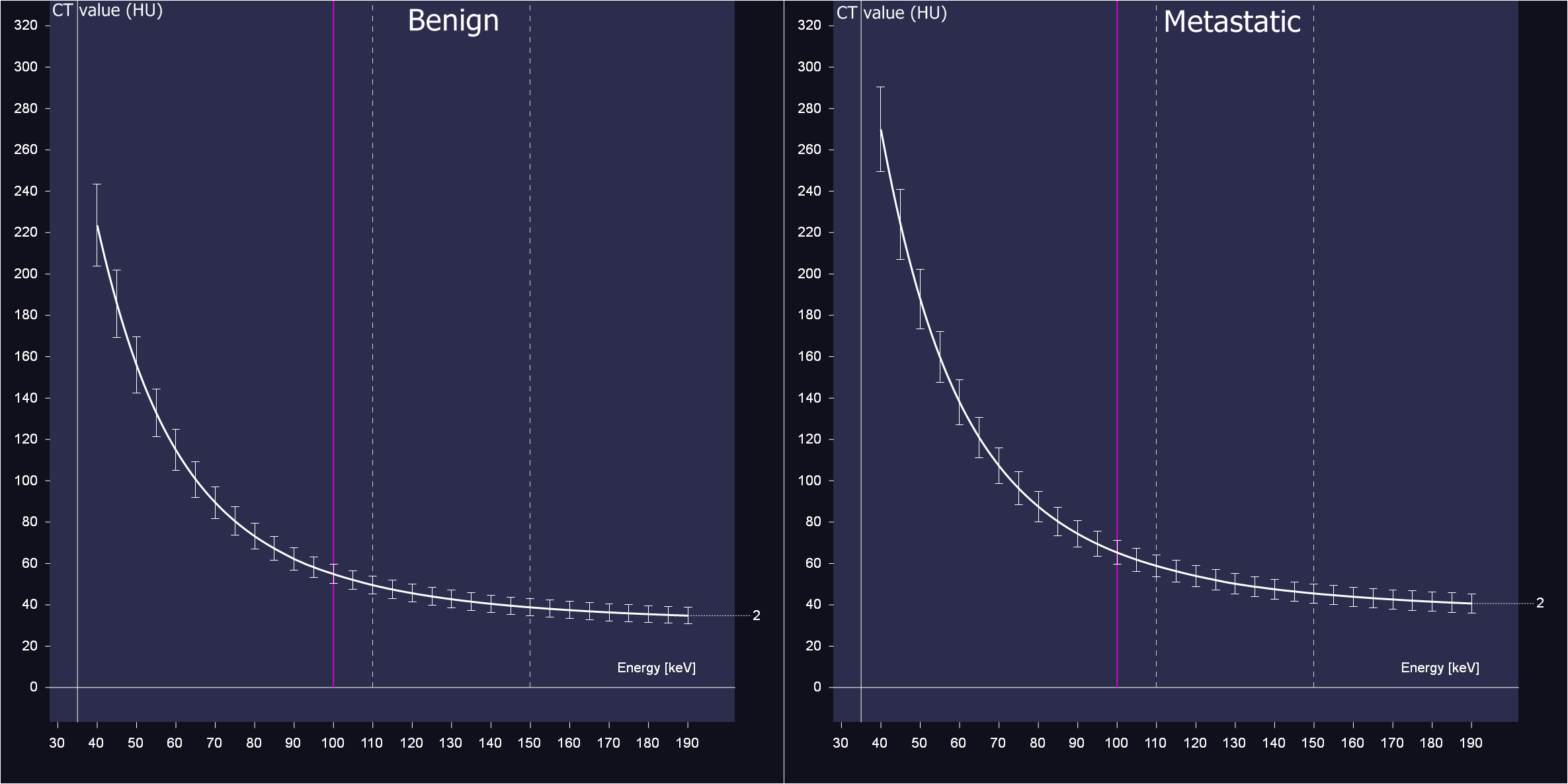

Supplement: S2 Fig — (TIF) [file pone.0261233.s002.tif]
