# Supplementary material for: Preoperative assessment of cervical lymph node metastases in patients with papillary thyroid carcinoma: Incremental diagnostic value of dual-energy CT combined with ultrasound
Source: PLoS One. 2021 Dec 13;16(12):e0261233. doi: 10.1371/journal.pone.0261233 (PMC8668122; doi:10.1371/journal.pone.0261233)
Supplement: S1 Table — (DOCX) [file pone.0261233.s003.docx]

**Supporting Table 1.** Comparison of DECT-derived parameters between the two methods of ROI measurements

|  | ROI with largest cross-sectional area (n=130) | Average of three ROIs (n=65) | *P*-value |
| --- | --- | --- | --- |
| arterial_norm | 0.31 ± 0.11 | 0.31 ± 0.12 | 0.999 |
| mono_40_norm | 0.53 ± 0.16 | 0.53 ± 0.17 | 0.969 |
| mono_70_norm | 0.58 ± 0.16 | 0.58 ± 0.17 | 0.829 |
| rho_norm | 0.74 ± 0.26 | 0.74 ± 0.26 | 0.918 |
| z_norm | 0.89 ± 0.04 | 0.90 ± 0.05 | 0.729 |
| vnc_norm | 0.77 ± 0.31 | 0.84 ± 0.44 | 0.267 |
| cm_norm | 0.45 ± 0.19 | 0.45 ± 0.20 | 0.919 |
| mixed_norm | 0.53 ± 0.18 | 0.55 ± 0.19 | 0.636 |
| iodine_norm | 0.43 ± 0.18 | 0.44 ± 0.18 | 0.687 |
| slope | 3.54 ± 1.55 | 3.41 ± 1.50 | 0.604 |
