# Supplementary material for: Preoperative assessment of cervical lymph node metastases in patients with papillary thyroid carcinoma: Incremental diagnostic value of dual-energy CT combined with ultrasound
Source: PLoS One. 2021 Dec 13;16(12):e0261233. doi: 10.1371/journal.pone.0261233 (PMC8668122; doi:10.1371/journal.pone.0261233)
Supplement: S2 Table — (DOCX) [file pone.0261233.s004.docx]

**Supporting Table 2.** Intraclass correlation coefficients of DECT-derived parameters acquired by two methods of ROI measurements

|  | Intraclass correlation coefficient |
| --- | --- |
| arterial_norm | 0.988 |
| mono_40_norm | 0.961 |
| mono_70_norm | 0.966 |
| rho_norm | 0.936 |
| z_norm | 0.967 |
| vnc_norm | 0.64 |
| cm_norm | 0.984 |
| mixed_norm | 0.979 |
| iodine_norm | 0.951 |
| slope | 0.963 |
